# Malaria Outbreak Facilitated by Appearance of Vector-Breeding Sites after Heavy Rainfall and Inadequate Preventive Measures: Nwoya District, Northern Uganda, February–May 2018

**DOI:** 10.1155/2020/5802401

**Published:** 2020-04-22

**Authors:** Godfrey Nsereko, Daniel Kadobera, Denis Okethwangu, Joyce Nguna, Damian Rutazaana, Daniel J. Kyabayinze, Jimmy Opigo, Alex R. Ario

**Affiliations:** ^1^Uganda Public Health Fellowship Program, Ministry of Health, Kampala, Uganda; ^2^National Malaria Control Division, Ministry of Health, Kampala, Uganda

## Abstract

**Background:**

Malaria is a leading cause of morbidity and mortality in Uganda. In April 2018, malaria cases surged in Nwoya District, Northern Uganda, exceeding expected limits and thereby requiring epidemic response. We investigated this outbreak to estimate its magnitude, identify exposure factors for transmission, and recommend evidence-based control measures.

**Methods:**

We defined a malaria case as onset of fever in a resident of Anaka subcounty, Koch Goma subcounty, and Nwoya Town Council, Nwoya District, with a positive rapid diagnostic test or microscopy for malaria from 1 February to 25 May 2018. We reviewed medical records in all health facilities of affected subcounties to find cases. In a case-control study, we compared exposure factors between case-persons and asymptomatic controls matched by age and village. We also conducted entomological assessments on vector density and behavior.

**Results:**

We identified 3,879 case-persons (attack rate [AR] = 6.5%) and two deaths (case-fatality rate = 5.2/10,000). Females (AR = 8.1%) were more affected than males (AR = 4.7%) (*p* < 0.0001). Of all age groups, 5–18 years (AR = 8.4%) were most affected. Heavy rain started in early March 2018, and a propagated outbreak followed in the first week of April 2018. In the case-control study, 55% (59/107) of case-persons and 18% (19/107) of controls had stagnant water around households for several days following rainfall (OR_M-H_ = 5.6, 95% CI = 3.0–11); 25% (27/107) of case-persons and 51% (55/107) of controls wore full extremity covering clothes during evening hours (OR_M-H_ = 0.30, 95% CI = 0.20–0.60); 71% (76/107) of case-persons and 85% (91/107) of controls slept under a long-lasting insecticide-treated net (LLIN) 14 days before symptom onset (OR_M-H_ = 0.43, 95% CI = 0.22–0.85); 37% (40/107) of case-persons and 52% (56/107) of controls had access to at least one LLIN per 2 household members (OR_M-H_ = 0.54, 95% CI = 0.30–0.97). Entomological assessment indicated active breeding sites in the entire study area; *Anopheles gambiae sensu lato* species were the predominant vector.

**Conclusion:**

Increased vector-breeding sites after heavy rainfall and inadequate malaria preventive measures were found to have contributed to this outbreak. We recommended increasing coverage for LLINs and larviciding breeding sites in the area.

## 1. Background

Malaria is a febrile illness caused by infection with the parasite *Plasmodium malariae, vivax, ovale,* and *falciparum* species. In Uganda, malaria remains a leading cause of morbidity and mortality, accounting for 13 million episodes annually, half of outpatient visits, and a third of hospital admissions nationally [1]. In 2017 alone, the World Health Organization estimated that Uganda had 11,700,000 confirmed indigenous cases and 5,100 reported malaria deaths [2]. Malaria transmission in Uganda occurs in over 95% of the country. The remaining 5% of the country consists of unstable and epidemic-prone transmission areas in the highlands of the south- and mid-west, along the southern border with Rwanda, and the northeastern border with South Sudan [1]. In Uganda, the predominant *Plasmodium* species is *P. falciparum,* accounting for 99% of cases, according to the Uganda Malaria Indicator Survey, 2014 [3]. The malaria vectors are mosquitoes of the *Anopheles* family, which breed in fresh water and temporary pools, such as those left by footprints and small ditches in the road. This can be especially true after rainfall and irrigation activities [3].

Uganda has made tremendous progress in malaria control with parasitemia in children under five years reduced from 42% in 2009 to 19% in 2014, while mortality due to malaria reduced from 20,000 persons in 2005 to about 5,000 in 2016. This reduction is a result of substantial investments in malaria control over the years, leading to increased access to key interventions such as long-lasting insecticide-treated nets (LLINs), indoor residual spraying (IRS), and antimalarial therapies [3].

The northern region of Uganda, where Nwoya District is located, has experienced malaria outbreaks during the last decade and has been associated with seasonal variations in the region [4, 5]. As a result, the region has been targeted for integrated vector control interventions, including IRS and distribution of LLINs. The most recent round of IRS in Nwoya District was in February 2017, while the last mass LLIN distribution occurred in early 2018 [5, 6].

**Figure 1:**
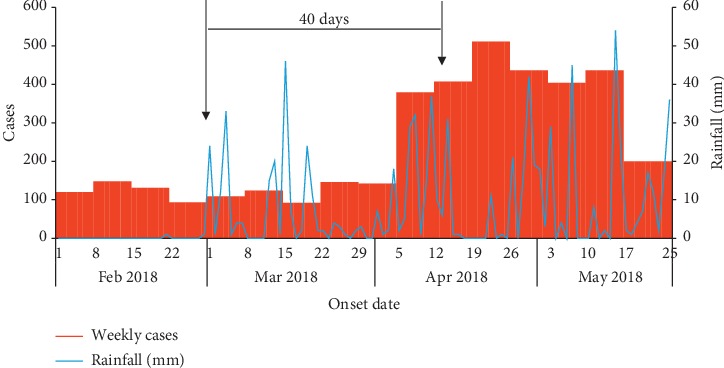
Weekly cases and daily rainfall data during a malaria outbreak in Nwoya District, Uganda, February–May 2018. Weekly confirmed cases (red bar graph) surged 40 days after start of rains (blue line graph). Confirmed cases reduced from outbreak levels to normal levels by mid-June 2018.

**Figure 2:**
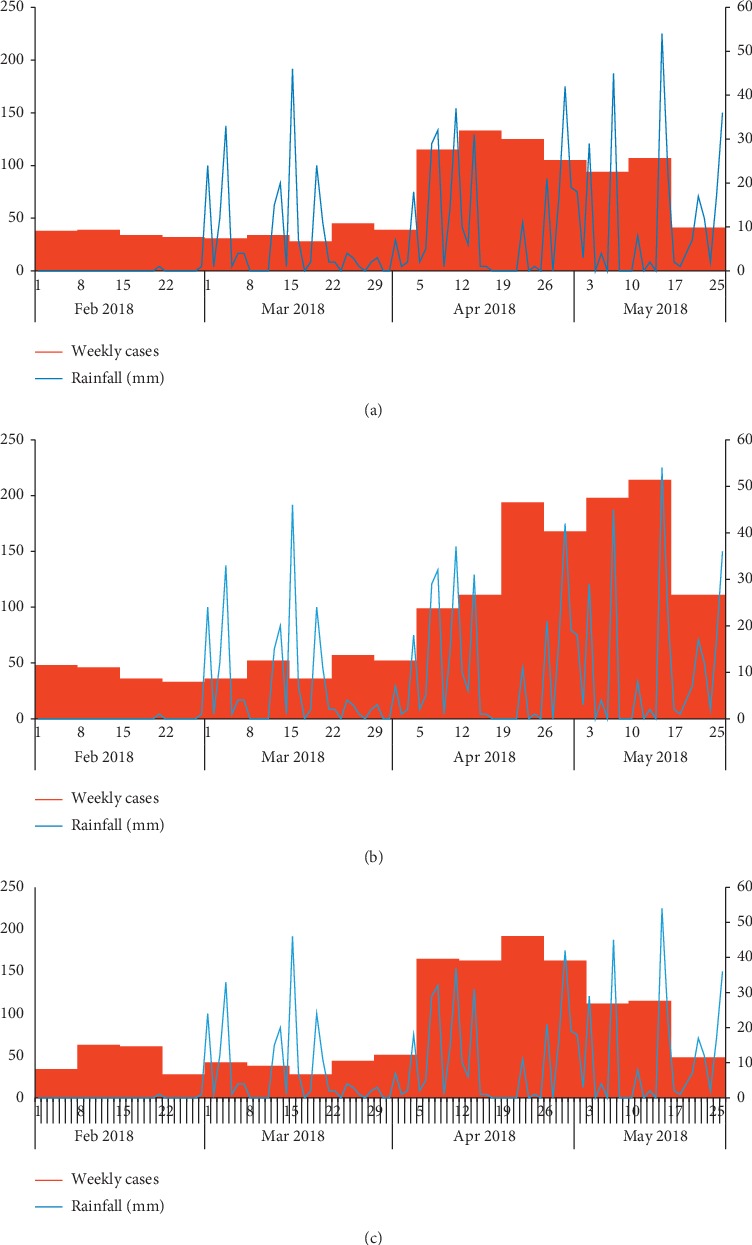
Weekly cases and daily rainfall data during a malaria outbreak, stratified by affected subcounty in Nwoya District, Northern Uganda, February–May 2018. Anaka subcounty (a), Koch Goma subcounty (b), and Anaka Town Council (c). The three subcounties received equal amounts of rainfall during the period.

**Figure 3:**
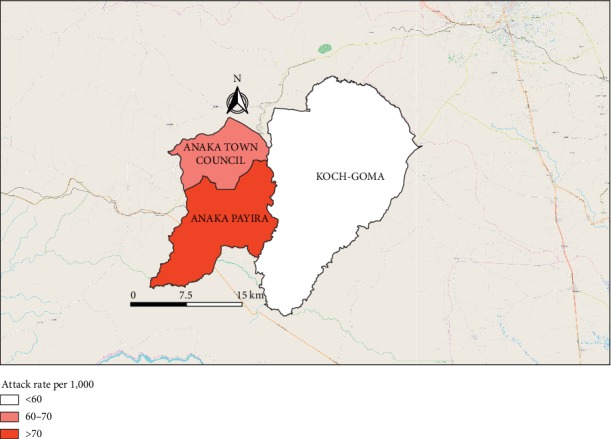
Map of 3 affected subcounties during a malaria outbreak in Nwoya District, Northern Uganda, February–May 2018. Anaka subcounty (labelled Anaka Payira) was the most affected subcounty, followed by Anaka Town Council.

On 18 April 2018, the district health officer (DHO) of Nwoya District reported an upsurge in the number of malaria cases in the district. Normal channel graphs for malaria cases constructed with data from the District Health Information System (DHIS2) showed that malaria cases had exceeded outbreak thresholds in 3 subcounties (Anaka subcounty, Koch Goma subcounty, and Nwoya Town Council). The outbreak thresholds had been established using malaria trends for preceding 5 years at district level. We therefore investigated this outbreak to determine the magnitude of the problem, identify risk factors for transmission, and recommend evidence-based control measures (Figures 1–3).

## 2. Methods

### 2.1. Study Area

Nwoya District (02° 38′N, 32° 00′E) is located in Acholi subregion, northern Uganda. The district is bordered by 6 districts: Masindi to the south; Buliisa to the southwest; Kiryandongo to the southeast; Oyam to the east; Gulu to the northeast; and Amuru to the north. The district is located at an altitude of 3,220 feet above sea level and experiences both wet and dry seasons. The wet season lasts from March to November and is warm and humid, while the dry season is hot and lasts from December to February. Rainfall peaks are experienced in May, June, August, and October. All year round, the temperature varies between 18 degrees Celsius and 36 degrees Celsius [7].

Administratively, Nwoya District comprises one county and 5 subcounties. The district is predominantly rural with a population of 133,506 people, a population density of 37 people per square kilometer, and projected population growth rate of 10% per year [8]. The district has one general hospital, three health center (HC) III and 14 HC II. All the health facilities have capacity to test for and treat malaria. The ownership of at least one mosquito net per household is 89% [8].

### 2.2. Case Definition and Finding

We defined a confirmed case as a positive malaria result by the histidine-rich protein II rapid diagnostic test (mRDT) or microscopy in a resident of Anaka subcounty, Koch Goma subcounty, or Nwoya Town Council, Nwoya District, from 1 February 2018 to 25 May 2018. We systematically searched for malaria cases by reviewing outpatient health records in all health facilities in the three affected subcounties. Malaria cases diagnosed by community health workers were captured from the attached health facility. We abstracted case-patient information on age, sex, village, parish and subcounty of residence, date of fever onset, diagnostic test done, and the test result. We line listed all the case-patients who fit the case definition.

### 2.3. Descriptive Epidemiology

We constructed epidemic curves (overall and subcounty stratified curves) to assess time distribution of malaria cases during the study period. We superimposed a line graph showing rainfall data over the same period. Using population data obtained by extrapolating 2014 Uganda National Population Census, Nwoya District-specific population growth rates, we computed attack rates by person (age group and sex) and place (parish and subcounty) characteristics [8].

### 2.4. Hypothesis Generation

In order to develop viable working hypotheses on possible contributors to the malaria outbreak, we conveniently sampled and interviewed 32 confirmed case-patients in the two most affected villages. We also observed their household environments for active and potential mosquito breeding sites and other possible exposures that could be associated with malaria transmission. The exposure variables explored before symptom onset were as follows: net ownership and use, patient activity and behavior during evening hours, wearing full extremity covering clothes in evenings, having standing water around households following heavy rains, and presence of screening curtains on doors and windows in evenings.

### 2.5. Case-Control Study

To test the hypotheses developed based on the descriptive epidemiology and the hypothesis generation, we conducted a case-control study in the three affected subcounties. We defined a control as an asymptomatic resident of Anaka subcounty, Koch Goma subcounty, or Nwoya Town Council, Nwoya District, from 1 February 2018 to 25 May 2018 with no evidence of positive malaria test result, at least 4 weeks from the date of symptom onset in the case-patient. We selected cases and controls for the study using probability proportional to size sampling [9]. In this method, the number of cases and controls sampled from each village included in the case-control study was proportional to the malaria attack rate of the village, with highest number of cases and controls selected from most affected villages. We chose case-patients and corresponding controls from seven villages that contributed 60% of the case-patients during the outbreak. We conducted systematic sampling among all households that had a confirmed malaria case. Basing on the number of households identified, we picked a case from every 6th case-household to be included in the case-control study. For controls, we conducted systematic sampling among all households that had no case but asymptomatic controls. Basing on the number of households identified, we decided to pick a control from every 10th control household. We administered a pretested questionnaire to each case-person and control. The parameters assessed in the questionnaire included sociodemographics, clinical presentation of cases at time of illness, LLIN use and ownership, and malaria transmission factors. For case-persons or controls who were minors, we administered the questionnaire to caregivers or guardians. For each case-person, we selected one asymptomatic control individually matched by village and age (±24 months).

### 2.6. Environmental Assessment

Based on the descriptive epidemiology, we walked through the most affected villages to examine environmental and human factors that may have catalyzed the upsurge in malaria cases during the period. We used an observational checklist for environmental assessment. The environmental factors assessed were presence and proximity to swampy areas, bushes, and other residual breeding sites for mosquito vectors. Human factors assessed was presence and proximity to human activity that potentiated mosquito breeding such as swamp farming, brick laying, and land excavation. We also identified both active and potential breeding sites.

### 2.7. Entomological Assessment

We assessed the vector density by randomly selecting homesteads within affected villages and conducting pyrethrum spray catches (PSC, a method that collects indoor resting mosquitoes by spraying a pyrethrum insecticide in the space and collecting mosquitoes that are knocked down on a white sheet laid on the ground). The collected mosquitoes were picked using forceps and identified based on morphological features on their legs, wings, and pulps using an identification key [10]. Pyrethrum spray catches were done daily in randomly selected houses between 24 and 28 May 2018, from 6 : 00 am to 10 : 00 am. We also conducted larval scoops in swamps and water-logged areas to identify both active and potential breeding sites.

To compute indoor resting density (IRD) for vectors, we calculated using the following formula:

(1) $$\begin{array}{c} \text{IRD }=\;\frac{\text{no. of mosquitoes captured indoors}/\text{no. of households}}{\text{no. of nights}}. \end{array}$$

To compute larval density in breeding sites around sampled households, we used the household and container indices using the formulae below:

(2) $$\begin{array}{c} \text{house index }=\;\left(\frac{\text{houses found positive with larvae}}{\text{total houses searched}}\right)\times 100,\\ \text{container index }=\;\left(\frac{\text{containers found positive with larvae}}{\text{total containers searched}}\right)\times 100\text{.} \end{array}$$

### 2.8. Data Analysis

We entered data into Microsoft Excel and exported them to Epi Info 7.2.2.0 software for analysis. We calculated frequencies and proportions for categorical variables, and means and medians for continuous variables. We analyzed outcome variables against possible exposures. For the case-control study, we developed case-control sets for analysis and obtained Mantel–Haenszel odds ratios. We used the chi-square test to establish differences among categorical variables and groups. A *p* value < 0.05 was taken as sufficient evidence to reject the null hypothesis of no difference. The magnitude of association and degree of uncertainty was calculated with odds ratios with 95% confidence intervals. We used a deductive approach to theme, summarize, and analyze data from the environmental assessment observations.

## 3. Results

### 3.1. Descriptive Epidemiology

We identified 3,879 case-patients (overall attack rate [AR] = 65/1000) including 2 deaths (case-fatality rate [CFR] = 0.05%). The deaths occurred in children <5 years old. The median age of the case-patients was 11 years (range 1 month to 90 years). The most affected age groups were children <5 years (AR = 69/1000) and 5–18 years (AR = 84/1000). Females (AR = 81/1000) were more affected compared to males (AR = 47/1000) (Table 1). The epidemic curve indicated a propagated outbreak with cases upsurging in late March 2018 after start of heavy rains in Nwoya District. The increase in malaria cases in excess of outbreak action thresholds began about 40 days from onset of heavy rainfall (Figure 1 and 2). The Anaka subcounty was the most affected (AR = 84/1,000) (Figure 3).

### 3.2. Hypothesis Generation Findings

Ninety-one percent (29/32) of case-patients slept in mud-walled houses, 84% (27/32) had standing water around their homesteads 3–5 days following heavy rainfall, 69% (22/32) were surrounded by overgrown bushes, 62% (20/32) did not sleep under an LLIN prior to symptom onset, and 66% (21/32) had a sick household member at the time of illness. Based on the descriptive epidemiology and hypothesis generation findings, we hypothesized that inconsistent use of LLINs and presence of nearby breeding sites after rainfall facilitated the malaria outbreak.

### 3.3. Entomological Assessment

Morphological investigation indicated that malaria vectors *Anopheles gambie sensu lato* and *Anopheles funestus* were the most common vectors. The average indoor resting density (IRD) of malaria vectors was ∼4 mosquitoes per household per night. The IRD of *Anopheles gambiae sensu latu* species was 4.6 mosquitoes/household/night, while that of *Anopheles funestus* species was 2.8. During larval density assessment, we searched 214 households and found 29 containers. The larval density in breeding sites around sampled households showed a household index of 2.3% and container index of 17.2%.

### 3.4. Environmental Assessment

Following the start of heavy rains in early March 2018, there was flooding of the River Akago, which flows through Anaka Town Council and proximal to Akago Parish. Another swamp around the market area in the town council temporarily flooded during the heavy rains. We found mosquito larvae in the flooded areas; these areas took 3 weeks to dry up, due to the ongoing rains. In the Anaka subcounty, an area that had been excavated for soil mining also served as a reservoir for stagnant water. We found active breeding sites for mosquitoes in the excavated area.

### 3.5. Case-Control Study Findings

In the case-control study, the coverage of adequate LLINs (one for every two household members) was 37% (40/107) among cases and 52% (56/107) among controls (OR_M-H_ = 0.54, 95% CI = 0.3–0.97). 71% (76/107) of case-persons and 85% (91/107) of controls slept under a long-lasting insecticide-treated net (LLIN) 14 days before symptom onset (OR_M-H_ = 0.43, 95% CI = 0.22–0.85); 37% (40/107) of case-patients and 52% (56/107) of controls had ≥1 LLIN per 2 household members (OR_M-H_ = 0.54, 95% CI = 0.30–0.97); 55% (59/107) of case-patients and 18% (19/107) of controls had stagnant water around households for 3–5 days following rainfall (OR_M-H_ = 5.6, 95% CI = 3.0–11.3); 65% (70/107) of case-patients and 36% (39/107) of controls did not use door and window curtains during evenings to minimize mosquito entry into the house (OR_M-H_ = 3.3, 95% CI = 1.8–6.0); and 25% (27/107) of case-patients and 51% (55/107) of controls wore full extremity covering clothes during evening hours both indoors and outdoors (OR_M-H_ = 0.30, 95% CI = 0.20–0.60) (Table 2).

## 4. Discussion

Our study highlights that malaria remains a big public health problem in malaria-endemic Uganda, even in the presence of robust malaria prevention and control interventions in areas like Nwoya District in northern Uganda where LLIN ownership stands at 89%. Also, the study points out that focusing on supplementary-integrated approaches towards malaria prevention and control can curb transmission in malaria-endemic areas. We found that having stagnant water near the home for 3–5 days following heavy rainfall, not sleeping under a LLIN, and absence of curtains on house doors and windows contributed to the outbreak. Conversely, we found that wearing full extremity covering clothing in evenings when indoors or outdoors was protective against malaria infection during the outbreak period.

Nwoya District was part of the many districts in northern Uganda where a malaria epidemic occurred in 2015 [5, 6]. Malaria epidemics have been reported since the discovery of the disease and its mode of transmission as a vector-borne disease more than 100 years ago [11]. As early as the 1950s, malaria outbreaks with devastating effects were reported in Ethiopia, Africa [12]. Outbreaks of malaria have been reported in China with specific interest on risk factors for transmission in the affected areas [13, 14]. In neighboring Burundi, a malaria outbreak has affected more than five million people since the start of 2019 [15]. In Madagascar, a malaria epidemic prevention was so critical in 2014 that a web-based application was used for malaria early warning systems [16]. The ubiquitous occurrence of malaria outbreaks necessitates institution of early warning systems for early detection and response to malaria outbreaks. In Uganda currently, the Uganda National Malaria Control Program works closely with meteorological authorities to strengthen systems for malaria epidemic early detection.

Nwoya District has over the past 5 years before the study experienced heavy rainfalls during the period from March to May [17, 18] when peak rainfall is received in the area. During the rainfall periods, temporary pools of standing water formed in areas with swamps, ditches, and open containers/vessels. Similarly, human activity such as soil excavation and road construction created temporary pools of stagnant water that provided active breeding sites for mosquitoes and facilitated transmission. Living in areas near stagnant water has been reported to increase risk of malaria infection in other studies [19, 20]. Studies indicate that rainfall has a positive seasonal association with malaria transmission, with mosquito populations observed to increase when rainfall increases to certain thresholds. In Kwa-Zulu Natal, South Africa, a study designed to assess rainfall variability on malaria transmission dynamics reported that malaria burden increased with increasing mean monthly rainfall in the range of 32–110 mm of rainfall [21]. In South Sudan, another study indicated that mosquito populations, and hence malaria transmission, increased when mean rainfall was in the range of 20–30 mm [22]. A study in arid and semiarid regions of Kenya reported a time lag of 1–2 months between rainfall and increase in malaria transmission [11]. This is consistent with 40 days' time lag that we found in our study. This rainfall-transmission time lag reflects the need for mosquito populations to grow before malaria transmission can increase. Therefore, prevention and control measures should be instituted when the first early warning signs (of increased rainfall) occur in epidemic-prone areas.

The use of LLIN for protection against human-vector contact during sleep is widely accepted as effective and largely embraced in Uganda [4, 23, 24]. Provision of LLIN to all populations ensures optimal protection against human-vector contact during sleep, when utilized correctly and consistently. In Kenya, a study reported a more than 30% decrease in hospitalization of children under 5 years of age following increased LLIN use [25]. Another study in Liberia reported that urban populations are more likely to benefit from LLIN use compared to rural populations owing to difference in population density factors [26]. Despite the reported positive gains of LLIN use, many other studies report no significant difference in malaria transmission between bed net users and nonbed net users. One observational study conducted in Haiti found no significant reduction in clinical malaria following mass LLIN campaigns [27]. A study in Tanzania cited underlying reasons for very high bed net use and corresponding high malaria infection prevalence among bed net users as poor sleeping habits, bed net users entering bed at different times, and going to bed late after being bitten by mosquitoes [28]. Some studies have cited change in behavior of mosquito vectors and biting victims before they enter bed nets at night [29]. Some studies have indicated that nets were not even utilized when available for various reasons, such as discomfort due to heat [30].

The availability of one LLIN for every two household members in our study was low, compared to national levels (65%) reported in the 2017 midterm review of the malaria reduction strategic plan [31]. Therefore, whereas LLIN use strategy is pivotal in prevention of malaria transmission, it should be used with an integrated approach with other malaria prevention and control strategies for optimal outcomes.

Whereas our study identified children between 5 and 18 years as the most affected, children under 5 years of age are the main victims of malaria in Uganda and worldwide [2, 3, 32]. A secondary data analysis of the Uganda Malaria Indicator Survey of 2014 reported an increasing malaria parasite prevalence among children under 5 years associated with severity of anemia [33]. Studies in Ethiopia [34], Nigeria [35], and Mali [36] reported the increased focus of malaria prevention strategies to children under 5 years as being the most affected. In a community level study in Malawi, children under 5 years and those between 5 and 19 years were reported to benefit equally from community bed net coverage [37]. We found another study in the Gambia that reported similar findings to our study [38]. It is possible that children <5 years were less affected than the 5–18 age group because the children are given priority by their guardians to sleep under LLINs, compared with older children. Even at national strategic level, this phenomenon is depicted where children under 5 years are targeted for chemoprevention [35, 36] and targeted bed net distribution [25–27].

We found that using cost-effective interventions such as clearing breeding sites, wearing long extremity clothing, and using curtains and screens on doors and windows can curb malaria transmission in outbreak situations. These approaches supplement WHO-recommended Integrated Vector Management (IVM) strategies in the fight against malaria [39]. Personal protection to prevent human-vector contact is effective where vectors feed on only humans but may become ineffective where mosquitoes feed on both human and animal populations [40]. Wearing clothes that are long enough to cover arms and legs and stockings to cover feet can provide significant protection against malaria in the evenings, as our study findings indicate. Insecticide-treated clothing has also been provided to outdoor workers in various setting to protect them against mosquito bites [41, 42]. In a study in Nigeria, protective clothing given to children under 5 years was reported to increase protection against malaria infection and anemia status [43]. We recommended to the National Malaria Control Program to incorporate integrated approach messages about behavior change communication to the general public.

Our findings in this study point to no significant protection against malaria in households that had been sprayed with indoor residual spraying (IRS) in 2017. This is not entirely surprising, as the potency of the insecticide sprayed in February 2017 would have diminished 6–9 months after spraying. A study in northern Uganda reported that whereas malaria prevalence reduction was realized within 3 months of IRS, the effects started to wane by the fourth month [44]. Another study in Zambia reported that IRS effectiveness using a specific insecticide was more effective during the rainy season than the dry season [45]. Therefore, whereas IRS has positive gains in reducing malaria transmission, it should be applied consistently to sustain such gains. This is at the center of the implementation of the Uganda National Malaria Reduction Strategic Plan, which has emphasized IVM as one of the key strategies to fast track malaria elimination [1, 46]. Strategies including source reduction, environment management, personal protection, and chemical control need to be applied in contextualized proportions to fend off malaria infection [47]. Following this study, we recommended larviciding of vector-breeding sites and increasing coverage and use of LLINs in the affected areas.

### 4.1. Study Limitations

The findings of the investigation were for only three subcounties in Nwoya District and may therefore not be generalizable to the whole district. In addition, some case-persons may have reported multiple times to the health facility for testing and treatment for the same malaria episode. This could have been due to nonadherence to medication given at the first attendance, or to reinfection. The former could lead to overestimation of the magnitude of the outbreak.

## 5. Conclusions and Recommendations

Increased vector-breeding sites after heavy rainfall, together with inadequate malaria preventive measures, contributed to this outbreak. We recommended increasing coverage for LLINs and larviciding breeding sites. Also, district and local leaders should mobilize masses and create awareness on using full extremity covering clothing and door screens, under the Mass Action Against Malaria strategy of the Ministry of Health.

### 5.1. Public Health Actions

We removed abandoned and empty containers around households. We also sensitized community leaders and members on malaria prevention using cost-effective strategies such as consistent use of LLINs, closing windows and doors early, and wearing full extremity covering clothes.

## Figures and Tables

**Table 1:** Demographic characteristics of case-patients during a malaria outbreak: Nwoya District, Uganda, February–May 2018.

Characteristics		Population	Cases	% of cases (*N* = 3,879)	Attack rate/1,000
Age (years)	0–5	13402	926	24	69
	5–18	21533	1809	47	84
	>18+	25039	1,144	29	46

Sex	Females	30563	2485	64	81
	Males	29411	1394	36	47

Subcounty	Overall	59974	3879	100	65
	Anaka	12322	1040	27	84
	Town Council	21378	1348	35	63
	Koch Goma	26274	1491	38	57

**Table 2:** Exposure factors among case-patients and controls during malaria outbreak, Nwoya District, February to May 2018.

Exposure	% exposed			
	Case (*N* = 107)	Control (*N* = 107)	OR_M-H_	95% CI
Had standing water around HH for 3–5 days after rainfall	55	18	5.6^*∗*^	3.0−11.3
Had curtains on doors and windows in evenings	65	36	3.3^*∗*^	1.8−6.0
Slept under LLIN 2 weeks before symptom onset	71	85	0.43^*∗*^	0.22−0.85
Wore full extremity covering clothes in evening hours bothindoors and outdoors	25	51	0.3^*∗*^	0.2−0.6
Had at least one LLIN per 2HH member	37	52	0.54^*∗*^	0.3−0.97
Presence of empty abandoned containers around HH	13	6	2.5	0.9−8.4
Entered bed to sleep after 9 pm	64	60	1.2	0.7−2.1
Had overgrown bushes around HH	74	67	1.4	0.7−2.6
HH underwent IRS in 2017	45	49	0.9	0.5−1.5

^*∗*^Significant exposures; CI: confidence intervals; HH: household; IRS: indoor residual spraying.

## Data Availability

The data that support the findings of this investigation belong to the Uganda Public Health Fellowship Program but are available from the corresponding author upon reasonable request and with permission from the Uganda Public Health Fellowship Program.

## Abbreviations

ACT: Artemisinin combination therapy
AR: Attack rate
CI: Confidence interval
DHIS2: District Health Information Software 2
DHO: District Health Office
HH: Household
HMIS: Health Management Information System
IRS: Indoor residual spraying
IVM: Integrated vector management
LLIN: Long-lasting insecticide-treated bed nets
MAAM: Mass Action Against Malaria
MoH: Uganda Ministry of Health
NMCD: National Malaria Control Division
OR: Odds ratio
ORM-H: Mantel–Haenszel odds ratio
RDT: Rapid diagnostic test
PHFP: Uganda Public Health Fellowship Program
PSC: Pyrethrum spray catch
UBOS: Uganda Bureau of Statistics
WHO: World Health Organization.
